# Task compliance predicts suppression-induced forgetting in a large sample

**DOI:** 10.1038/s41598-021-99806-8

**Published:** 2021-10-11

**Authors:** Peiduo Liu, Justin C. Hulbert, Wenjing Yang, Yuhua Guo, Jiang Qiu, Michael C. Anderson

**Affiliations:** 1 Key Laboratory of Cognition and Personality (SWU), Ministry of Education, Chongqing, 400715 People’s Republic of China; 2 Faculty of Psychology, Southwest University, No. 2 TianSheng Road, Beibei District, Chongqing, 400715 People’s Republic of China; 3 Psychology Program, Bard College, Annandale-on-Hudson, NY 12504 USA; 4 MRC Cognition and Brain Sciences Unit, University of Cambridge, Cambridge, UK

**Keywords:** Psychology, Human behaviour

## Abstract

Suppression-induced forgetting (SIF) refers to a memory impairment resulting from repeated attempts to stop the retrieval of unwanted memory associates. SIF has become established in the literature through a growing number of reports built upon the Think/No-Think (TNT) paradigm. Not all individuals and not all reported experiments yield reliable forgetting, however. Given the reliance on task instructions to motivate participants to suppress target memories, such inconsistencies in SIF may reasonably owe to differences in compliance or expectations as to whether they will again need to retrieve those items (on, say, a final test). We tested these possibilities on a large (N = 497) sample of TNT participants. In addition to successfully replicating SIF, we found that the magnitude of the effect was significantly and negatively correlated with participants’ reported compliance during the No-Think trials. This pattern held true on both same- and independent-probe measures of forgetting, as well as when the analysis was conditionalized on initial learning. In contrast, test expectancy was not associated with SIF. Supporting previous intuition and more limited post-hoc examinations, this study provides robust evidence that a lack of compliance with No-Think instructions significantly compromises SIF. As such, it suggests that diminished effects in some studies may owe, at least in part, to non-compliance—a factor that should be carefully tracked and/or controlled. Motivated forgetting is possible, provided that one is sufficiently motivated and capable of following the task instructions.

## Introduction

Everyone has memories they might prefer to avoid—from mundane distractions to painful experiences of trauma or loss. One effective coping strategy may be to avoid potential reminders of the unwanted memories^1–3^. However, it is not always possible to predict when such reminders may appear, and habitual avoidance of cues deprives the person of the chance to develop strategies for coping with reminders when they do occur^4,5^. Adaptively controlling whether certain memories are retrieved when faced with reminders may afford certain mental health advantages, but not everyone is equally capable of effectively suppressing memory retrieval^6–10^. Recent work with the Think/No-Think (TNT) paradigm has focused on understanding the factors that give rise to such differences in effective control.

The TNT paradigm, introduced by Anderson and Green^11^, was developed to empirically test the ability to suppress unwanted memories by measuring a predicted aftereffect of repeated memory stoppage: suppression-induced forgetting. Participants undergoing this procedure typically are first asked to learn cue-target word pairs to criterion (e.g., 50%) before proceeding to the critical TNT phase of the experiment. At this stage, participants are reminded of a subset of the learned cue words and asked to retrieve their associated targets some number of times (Think condition). Another subset of learned reminders is instead presented with the instruction to suppress the associated target (No-Think condition).

The TNT paradigm reveals that the accessibility of memory associates is influenced by the manner in which individuals are instructed to handle repeated reminders. On the one hand, cueing individuals to retrieve the associated memory typically facilitates those Think items on later surprise recall tests. On the other hand, pairing No-Think instructions with reminders often yields suppression-induced forgetting (SIF)—impaired accessibility of the targets from the No-Think condition relative to those that were equally well learned but not cued during the TNT phase (i.e., Baseline items^10–12^). Such findings have been observed using a wide variety of methods and materials, including neutral and emotional stimuli, involving words, scenes, objects and even autobiographical memories^5,13–18^.

Notably, SIF often generalizes from cued-recall tests using the originally learned cue (the same-probe, SP, measure) to independent probes (IPs; e.g., either a semantic category cue with word stems; or a second, studied cue associated to the original response). Forgetting on the IP test is taken as a purer measure of the aftereffects of the suppression attempts on the targets commonly attributed to inhibition^19,20^. While other, non-inhibitory accounts of SIF have been considered^21^, a sizeable body of evidence continues to amass highlighting an inhibitory contribution^6,10^. SIF, for example, is notably absent in populations that are thought to suffer from deficits inhibitory control more generally, including older adults^19,22^ and the very young^23^. Similarly, individuals with attention-deficit/hyperactivity disorder (ADHD), depression, and dysphoria are known to sometimes exhibit diminished SIF^8^, a finding that tends to be further clarified by neuroimaging and electrophysiological techniques^24–26^. Additional evidence supporting the inhibition account stems from a growing number of neuroimaging studies of healthy adults pointing to a prefrontally mediated top-down inhibition of brain regions supporting aspects of the target memory representation^10^.

Despite numerous replications, behavioral evidence of SIF occasionally has failed to materialize^27–31^. Anderson and Huddleston^32^, among others, considered a number of factors that may have contributed to the lack of suppression-induced forgetting in such studies. In addition to fatigue, trial duration, and sleep deprivation^33,34^, variability in task compliance is a prime suspect. Indeed, SIF depends on both the capacity to suppress unwanted memories and, critically, the motivation to do so repeatedly, such that the aftereffects of memory control can be detected. In most cases, the relevant materials in the TNT paradigm are only “unwanted” in the sense that the experimenter-provided instructions designate them as such. In many cases, the target memories themselves are arbitrary and neutral in valence.

Without an inherent reason to suppress a memory (e.g., if the memory is particularly painful, disruptive, or embarrassing personally), some participants may decide to forgo the taxing challenge of stopping retrieval of what they had just been trained to bring to mind automatically. Indeed, some participants may even attempt to use the time during the lengthy TNT phase (for which overt responses are not typically required or collected) to check to make sure that they are still able to recall the materials—either as a way of keeping occupied without exerting too much effort or in an attempt to improve their memory for a final memory test they may have come to expect—this, despite the experimenters’ best attempts to keep the final test a surprise and ensure task compliance. As such, compliance would be expected to suffer, and SIF would, in turn, be expected to disappear or even reverse itself to become above-baseline facilitation of No-Think items. Hertel and Calcaterra^29^ found evidence for this intuition on the basis of a post-experiment compliance questionnaire: Only unaided participants who reported complying with the No-Think instructions exhibited a SIF trend; those who admitted to being uncompliant exhibited facilitation for the same items.

Most recent studies using the TNT paradigm have instituted a pre-established exclusion criterion on the basis of such a compliance questionnaire; however, systematic examinations of the effect of non-compliance remain limited. Robust evidence of a negative relationship between non-compliance and SIF would further substantiate such exclusions and the efforts undertaken to minimize them. The current study, powered with a large sample, allowed us to test for just such a relationship. We also considered whether the expectation of a final memory test is associated with increased non-compliance and reduced SIF.

## Method

### Participants

For Experiment 1, 146 participants (40 male and 106 female, all between 16 and 24 years of age) from the Southwest University in China participated as paid volunteers. Five participants were removed from the eventual data analysis as they asked to withdraw early from the study (before the final test) because they indicated becoming uncomfortable being in the MRI scanner for the full duration of the TNT phase, leaving 141 participants contributing to the analysis of SIF and facilitation effects. Three participants failed to complete the compliance questionnaire, while three did not complete the test-expectancy questionnaire because of experimenter error. These participants were included in the above analyses but were necessarily excluded from analyses involving the compliance and test expectancy. For Experiment 2, 351 (97 male and 254 female) undergraduate and postgraduate students (18–25 years of age) from the Southwest University in China participated. Five participants failed to complete the compliance questionnaire, while three failed to complete test-expectancy questionnaire; these participants were excluded from analyses involving compliance and test expectancy. Across both studies, all participants were right-handed native Chinese speakers with normal or corrected-to-normal vision. None reported a history of neurological or psychiatric disorders. All participants provided an informed consent prior to the study, which was approved by the Institutional Human Participants Review Board of Southwest University Imaging Center for Brain Research. The experimental procedures were approved by the Academic Committees of Southwest University in China and conducted in accordance with relevant guidelines and regulations.

### Design

The experiment used a 3 × 2 × 3 mixed-subjects design, with *Task* (Baseline, Think, and No-Think) and *Test Type* (Same-Probe vs. Independent-Probe test) manipulated within participants, and *Item Counterbalancing* manipulated between participants. We assessed the percentage of items correctly recalled on the final test as our primary dependent measure. We computed this measure in two ways: (1) based on all of the studied items, irrespective of whether they had been demonstrably learned prior to entering the TNT phase (*Unconditionalized recall data*); and (2) considering only those items that had been correctly recalled on the initial pre-test prior to the TNT phase (*Conditionalized recall data*). The primary analyses of the recall data focused on facilitation of Think items (Baseline vs. Think items) and suppression of No-Think items (Baseline vs. No-Think items).

We also measured participants’ compliance with the No-Think instructions using two post-experimental self-report questionnaires that have become standard in many implementations of the TNT paradigm^32^: a compliance questionnaire and a test-expectancy questionnaire (see below).

### Materials

We selected 66 weakly related word pairs from previous studies^11,12^ and translated these into Chinese. We divided the pairs into three subsets of 16, which were then separately assigned to the Think, No-Think, and Baseline conditions, counterbalanced across participants. We reserved the remaining 18 pairs as fillers to be used in the practice TNT phase and in the practice test used to reinstate the initial learning context before the critical final memory tests (see Procedure).

### Procedure

We used the conventional TNT paradigm^12^, which consists of three phases: a study phase, the TNT phase, and a final test phase. Both experiments used the same procedure, except that participants in Experiment 1 performed the TNT phase in the MRI scanner (hereinafter referred to as the fMRI sample), whereas participants in Experiment 2 performed the TNT phase outside of scanner (hereinafter referred to as the behavioral sample). We only used the behavioral data from these samples in this study; the imaging data were reported elsewhere^35^. In the sections below, we describe the details of each phase.

#### Study phase

In the study phase, we instructed participants to learn 66 cue-target word pairs so that they could recall the target as soon as they saw a cue word. The study phase took place in three stages. First, participants studied the word pairs one by one, with each word pair presented in a white font in the middle of a black screen for 3.4 s (0.6 s ITI). Second, participants had up to three test-feedback cycles to achieve at least 50% accuracy in recalling the associations. During this stage, we presented each cue word for up to 3.4 s (0.6 s ITI). After participants recalled the target word aloud or the 3.4 s had elapsed, participants viewed the correct target as feedback for 1 s. As a final step, we tested participants on all 66 pairs: We presented each cue word on the screen for 4 s and asked participants to report the target word aloud. This final “criterion test” was used to establish which pairs had been learned successfully, and performance on this test was used to decide which pairs would be analyzed in our conditionalized recall measure (see Design section).

#### Think/no-think phase

Although the TNT phase for Experiment 1 was conducted in the MRI scanner, rather than in a behavioral testing room, the TNT phase unfolded similarly across both experiments. Specifically, in this phase, each trial presented a single cue word from either the Think or No-Think conditions, which were randomly intermixed. We told participants that some of the cue words would appear in green (Think trials), and that their task for these items would be to recall the associated target as soon as possible and keep it in mind for the duration of the trial. In contrast, other cue words instead would appear in red (No-Think trials), and, for these trials, their task would be to prevent the associated target word from coming into awareness by blocking out all thoughts about it without replacing it with any other thoughts. As such, these No-Think instructions are consistent with the Direct Suppression technique described elsewhere^36^. During each trial, the cue word appeared for 3 s with a jittered ISI (1 s, 3 s, 5 s, 7 s) that helped optimize the efficiency of the event-related fMRI design. Participants viewed a fixation cross during the ISI. There was no jittered ISI in Experiment 2.

Before the TNT phase proper, we led participants through a practice TNT phase with fillers pairs to make sure participants fully understood and complied with the instructions at this stage. Not only are these efforts a standard part of what has become the TNT paradigm’s typical implementation, but we also wanted to establish that any reported non-compliance in the TNT phase proper was not due to a misunderstanding of the instructions at the outset of the task. The practice phase consisted of two short blocks. After each block, we administered a diagnostic questionnaire to ensure that participants understood the procedure, and we gave corrective feedback as necessary (e.g., if they covertly rehearsed the target words for No-Think trials or if they did not always actively push the target word out of mind if it did come to mind during the red cue). Participants received a 5-min break between the practice and the formal TNT phase. All the participants received a refresher presentation of all the word pairs before the TNT phase in the scanner (1 s per pair).

The TNT phase proper was divided into 6 blocks, each lasting for 6.7 min. Each block contained 16 Think items and 16 No- Think items pairs, with each item presented twice. We inserted a 30–40 s break after each block, and we administered an additional diagnostic questionnaire after the first three blocks to ensure that the participants continued to follow the instructions. We obtained the diagnostic questionnaire from Michael Anderson and translated it into Chinese.

#### Final test phase

We tested participants’ memory for the Think, No-Think, and Baseline items in two ways, in two separate test blocks, the order of which was counterbalanced across participants: a Same-Probe (SP) test and an Independent-Probe (IP) test. On each trial of the SP test block, we presented a cue word from one of the studied pairs on the screen for 3.4 s (ISI 0.6 s) and asked participants to recall aloud the word they had learned to associate with it during the initial study phase. On each trial of the IP test, in contrast, we presented a category or a semantically related cue of the target word on the screen for 3.4 s (ISI 0.6 s) and asked participants to recall a studied response word that fit those cues. Before these two tests, we administered to participants a practice test block of 18 filler word pairs containing a mixture of filler Baseline, Think, and No-Think items. This practice helped ensure participants understood the task procedure and also helped reinstate the context of the original study phase, in which they had learned the Baseline pairs, as well as the Think and No-Think pairs.

### Compliance questionnaire

After the memory tests, participants filled out a questionnaire to assess their compliance with the No-Think instructions. First, participants were asked to provide honest ratings from 0 (Never) to 4 (Always) on three statements to indicate whether they ever intentionally made an effort to think about the targets during No-Think trials: (a) When I saw the red cue word, I quickly checked to see if I remembered the target word; (b) After a red cue word went off the screen, I checked to see if I still remembered the target word; (c) When I saw a red cue word, I thought about the target word that went with it to purposely improve my memory for that word pair. We computed a summary compliance score across the three main “cheating” or “memory checking” behaviors (i.e., checking during the No-Think trial, checking after the No-Think trial, and intentional rehearsal of No-Think items). This score served as a key dependent measure that we used to examine a possible relationship between non-compliance and suppression-induced forgetting. We also analyzed these items separately.

### Test-expectancy questionnaire

Participants were then asked to indicate the extent to which they expected a final memory test. Specifically, they were asked to provide a rating from 0 to 4 (0 = “No, I did not think that”; 2 = “Unsure if I did think that”; 4 = “Yes, I definitely thought that”) in response to the following prompt:

In the main phase of the experiment, we asked you to not think about the associated target word for cue words colored in RED. During this phase, did you suspect that you would later be asked to recall the target for these RED cue words? In other words, did you anticipate some form of a final test?

## Results

### Training phase performance

For both experiments, all participants achieved the learning criterion of 50% within the three test-feedback cycles. For the fMRI sample, the mean recall percentage (and standard deviation) on the criterion test at the end of the learning phase was 0.78 ± 0.14. The recall percentages of items in the Think (*M* = 0.80 ± 0.16), No-Think (*M* = 0.78 ± 0.16), and Baseline conditions (*M* = 0.76 ± 0.16) were not significantly different, *F* (2, 420) = 1.142, *p* > 0.05. For the behavioral sample, the mean recall percentages for the Think (*M* = 0.85 ± 0.15), No-Think (*M* = 0.83 ± 0.14), and Baseline conditions (*M* = 0.83 ± 0.15) were also not significantly different, *F* (2, 1052) = 1.278, *p* > 0.05.

### Final test phase performance

To examine the SIF effect, we conducted a *Task* (Baseline vs. No-Think) by *Test Type* (Same-Probe vs. Independent-Probe test) repeated-measures ANOVA for the percentage of items correctly recalled. To examine facilitation of practiced items, we conducted a *Task* (Think vs. Baseline) by *Test Type* (Same-Probe vs. Independent-Probe test) repeated-measures ANOVA. Because we used the same experimental design in the two experiments, we first combined data from both to increase the statistical power and to establish the general pattern of results. We followed this by an analysis of the two experiments reported separately. In addition, we report the foregoing analyses both using the conditionalized and the unconditionalized recall data (see Design). We present the mean recall percentage for each condition for each sample in Table 1.

**Table 1:** Final recall accuracy on the Same-Probe (SP) and the Independent-Probe (IP) tests.

Condition	Baseline	No-think	Think
**Experiment 1 (fMRI)**			
SP test			
Conditionalized	89% [87, 91]	83% [80,86]	92% [90, 93]
Unconditionalized	76% [73, 79]	72% [68, 75]	81% [78, 83]
IP test			
Conditionalized	55% [52, 58]	45% [42, 48]	43% [40, 46]
Unconditionalized	47% [44, 50]	40% [37, 42]	39% [36, 41]
**Experiment 2 (behavioral)**			
SP test			
Conditionalized	90% [89, 91]	78% [76, 81]	94%[93, 95]
Unconditionalized	78% [77, 80]	69% [66, 71]	85% [83, 87]
IP test			
Conditionalized	53% [51, 55]	46% [44, 48]	47% [45, 49]
Unconditionalized	49% [47, 51]	43% [41, 45]	44% [43, 46]
**Full sample**			
SP test			
Unconditionalized	78% [76, 79]	70% [68, 71]	84% [82, 85]
Conditionalized	90% [89, 91]	80% [78, 82]	93% [92, 94]
IP test			
Unconditionalized	48% [47, 50]	42% [41, 43]	43% [41, 44]
Conditionalized	54% [52, 55]	46% [44, 48]	46% [44, 47]

Values in brackets reflect the 95% confidence interval for the marginal means.

### Suppression impaired memory performance

Across both experiments, we observed a significant main effect of *Task* in the conditionalized data, *F*(1, 491) = 156.751, *p* < 0.001, *η*_*P*_^*2*^ = 0.242, as well as in the unconditionalized data, *F*(1, 491) = 116.327, *p* < 0.001, *η*_*P*_^*2*^ = 0.192, indicating robust evidence for suppression-induced forgetting (SIF). For the fMRI sample, the main effect of *Task* also was significant in both the conditionalized data, *F*(1, 140) = 59.466, *p* < 0.001, *η*_*P*_^*2*^ = 0.298, and the unconditionalized data, *F*(1, 140) = 33.766, *p* < 0.001, *η*_*P*_^*2*^ = 0.194. Similarly, in the behavioral sample, the main effect of *Task* was significant in both the conditionalized data, *F*(1, 350) = 103.228, *p* < 0.001, *η*_*P*_^*2*^ = 0.228, and the unconditionalized data, *F*(1, 350) = 83.798, *p* < 0.001, *η*_*P*_^*2*^ = 0.193. Thus, all analyses showed that the overall SIF effect occurred when collapsed over *Test Type*, and this pattern did not depend on whether recall was conditionalized on correct initial learning or not.

The suppression-induced forgetting effect showed evidence of generalizing over both the Same-Probe and Independent-Probe tests, confirming the property of cue-independent forgetting thought to be critical evidence of inhibition. In the overall sample, there was no interaction between *Task* and *Test Type*, regardless of whether we examined the conditionalized data, *F*(1, 491) = 3.049, *p* > 0.05, *η*_*P*_^*2*^ = 0.006, or the unconditionalized data, *F* (1, 491) = 3.138, *p* > 0.05, *η*_*P*_^*2*^ = 0.006. In the conditionalized data, SIF was individually significant for the SP test, *F*(1, 491) = 107.007, *p* < 0.001, *η*_*P*_^*2*^ = 0.179, and the IP test, *F*(1, 491) = 65.722, *p* < 0.001, *η*_*P*_^*2*^ = 0.118; the same held true in the unconditionalized data (SIF for SP test: *F*(1, 491) = 78.043, *p* < 0.001, *η*_*P*_^*2*^ = 0.137; SIF for IP test: *F*(1, 491) = 57.044, *p* < 0.001, *η*_*P*_^*2*^ = 0.104).

The individual experiments showed largely similar patterns, although the amount of SIF did vary with *Test Type* in one of the samples. Within the fMRI sample, there was not a reliable interaction between *Task* and the *Test Type* in the conditionalized data, *F*(1, 140) = 2.237, *p* > 0.05, *η*_*P*_^*2*^ = 0.016, nor was there one within the unconditionalized data, *F*(1, 140) = 1.800, *p* > 0.05, *η*_*P*_^*2*^ = 0.013. In the behavioral sample, there was a significant interaction between *Task* and *Test-Type*, such that the SIF effect was larger on the SP test than it was on the IP test, *F*(1, 350) = 9.073, *p* < 0.005, *η*_*P*_^*2*^ = 0.025. Nevertheless, the simple effects showed significant SIF on both the IP test, *F*(1, 350) = 34.716, *p* < 0.001, *η*_*P*_^*2*^ = 0.090, and on the SP test, *F*(1, 350) = 89.513, *p* < 0.001, *η*_*P*_^*2*^ = 0.204. This pattern also held true in the unconditionalized data, which revealed a significant interaction of *Task* and *Test Type*, *F*(1, 350) = 8.430, *p* < 0.05, *η*_*P*_^*2*^ = 0.024, with significant SIF on both the IP test, *F*(1, 350) = 31.877, *p* < 0.001, *η*_*P*_^*2*^ = 0.083, and on the SP test, *F*(1, 350) = 71.416, *p* < 0.001, *η*_*P*_^*2*^ = 0.169. Thus, although one of the experiments revealed more SIF on the SP test than on the IP test, both experiments showed SIF that generalized over *Test Type*, consistent with cue-independence.

### Retrieval facilitated memory performance when tested with the trained cue

Across both experiments, we observed a significant main effect of *Task* (Baseline vs. Think) that arose in the conditionalized data, F(1, 491) = 16.752, *p* < 0.001, *η*_*P*_^*2*^ = 0.033, but not in the unconditionalized data, *F*(1, 491) = 0.160, *p* > 0.05, *η*_*P*_^*2*^ = 0.000. For the fMRI sample, the facilitation effect was significant in both the conditionalized data, *F*(1, 140) = 20.966, *p* < 0.001, *η*_*P*_^*2*^ = 0.130, and in the unconditionalized data, *F*(1, 140) = 5.007, *p* < 0.05, *η*_*P*_^*2*^ = 0.035. In the behavioral sample, facilitation was reliable in neither the conditionalized, *F*(1, 350) = 3.652, *p* > 0.05, *η*_*P*_^*2*^ = 0.010, nor the unconditionalized data, *F*(1, 350) = 2.892, *p* > 0.05, *η*_*P*_^*2*^ = 0.008. As such, the facilitation effect appeared to be less stable than the suppression effect reported above.

One reason for this lack of observed stability in the observed facilitation effect is that it interacted with *Test Type*. In the overall sample, the interaction of *Task* and the *Test Type* was significant in both the conditionalized data *F*(1, 491) = 108.474, *p* < 0.001, *η*_*P*_^*2*^ = 0.181, and the unconditionalized data, *F*(1, 491) = 134.704, *p* < 0.001, *η*_*P*_^*2*^ = 0.215*.* This effect arose in both the fMRI sample (conditionalized: *F*(1, 140) = 47.203, *p* < 0.001, *η*_*P*_^*2*^ = 0.252; unconditionalized: *F*(1, 140) = 53.471, *p* < 0.001, *η*_*P*_^*2*^ = 0.276) and the behavioral sample (conditionalized: *F*(1, 350) = 63.854, *p* < 0.001, *η*_*P*_^*2*^ = 0.154; unconditionalized: *F*(1, 350) = 83.768, *p* < 0.001, *η*_*P*_^*2*^ = 0.193). Examining the test types separately, the facilitation effect in the overall sample was significant on the SP test in both the conditionalized data, *F* (1, 491) = 42.104, *p* < 0.001, *η*_*P*_^*2*^ = 0.079, and the unconditionalized data, *F* (1, 491) = 104.032, *p* < 0.001, *η*_*P*_^*2*^ = 0.175. However, this effect was not significant for the IP test, and, indeed, was reversed in the unconditionalized data, *F* (1, 491) = 44.195, *p* < 0.001, *η*_*P*_^*2*^ = 0.083. Similar patterns arose in each individual experiment. These findings show that facilitation due to retrieval practice is entirely specific to the practiced association and does not generalize to independent cues. Indeed, retrieval practice can impair retention of a memory when it is tested via a novel cue, an effect sometimes observed in prior studies and which has been attributed to increasing encoding specificity due to retrieval practice^23^.

### Most participants reported complying with suppression instructions

**Figure 1:**
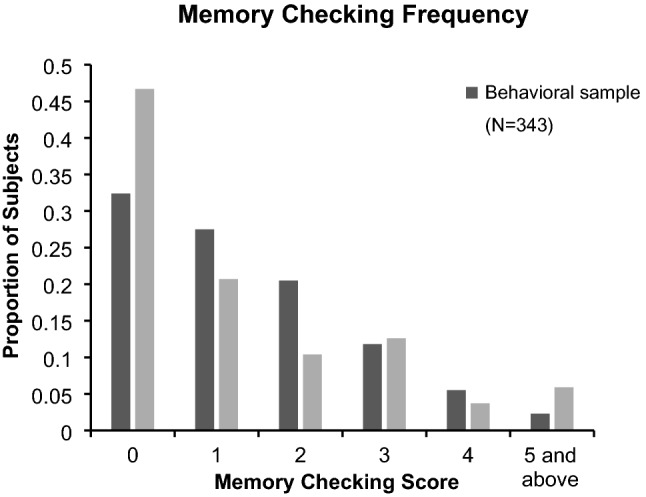
Proportion of participants reporting level of memory checking during and after the TNT trial in each of the samples; 0 = perfect compliance with the No-Think instructions as self-reported on the post-experiment compliance questionnaire.

Given that we provided participants with practice on the TNT task prior to the critical phase and gave them extensive feedback on the instructions for the No-Think task (via repeated administration of the diagnostic questionnaire), one might expect that most participants would report compliance with the No-Think instructions after the experiment. Consistent with this supposition, the results from our compliance questionnaire indicated that most participants at least claimed to be compliant with task instructions during the TNT phase (see Fig. 1). To get the non-compliance (memory checking) score, we summed the ratings across three non-compliance questions (each on a scale from 0 to 4). This yielded a score ranging from 0 to 12. The mean (± standard deviation) non-compliance score from the overall sample was 1.352 ± 1.424. For the fMRI sample and the behavior sample, the non-compliance scores were 1.259 ± 1.583 and 1.388 ± 1.388, respectively. So, although compliance was not perfect (which would be represented by a non-compliance score of exactly 0), participants did, on the whole, appear to avoid any intentional efforts to think of the No-Think items.

Nevertheless, non-compliance occurred in some cases. To characterize the nature of this behavior, we first examined the frequency of different types of non-compliance measured on the three relevant questions. For the overall sample, intentional checking of memory for No-Think items *during* a No-Think trial (the score of the first question in the compliance questionnaire) was more frequent than intentional checking just *after* the No-Think trial was over (the score of the second item of the compliance questionnaire), *t*(477) = 4.281, *p* < 0.001 (all significant *p* values in this set of analyses were Bonferroni corrected for repeated measurements). The results from the fMRI sample and the behavioral sample (respectively) also revealed this pattern individually, *t*(134) = 1.907, *p* < 0.05; *t*(342) = 3.836, *p* < 0.001.

Moreover, we found that checking *during* a trial was more likely than *intentional rehearsal* of the No-Think items (the rating for the third item of the compliance questionnaire) in the overall sample, *t*(477) = 14.128, *p* < 0.001. The data of the fMRI and behavioral samples (respectively) also showed this effect individually, *t*(134) = 5.793, *p* < 0.001; *t*(342) = 13.260, *p* < 0.001.

We also found that checking *after* a No-Think trial (the second item of the compliance questionnaire) was more likely than was intentional rehearsal (the third item of the compliance questionnaire) in the overall sample, *t*(477) = 10.193, *p* < 0.001. The results of the fMRI and behavioral samples individually showed that same pattern of results, *t*(134) = 4.653, *p* < 0.001; *t*(342) = 9.135, *p* < 0.001. Together, these results show that participants’ urge to quickly “check their memory” for No-Think items during or after a No-Think trial was more common than outright attempts to intentionally rehearse the items for the later test. This suggests that many people may view such “quick checks” of their memory for No-Think items as distinct from deliberate rehearsal of No-Think items, even though both behaviors are clear violations of the instruction to avoid awareness of the memory.

We had hypothesized that participants’ compliance in the fMRI sample would be higher than that of the behavioral sample because we repeatedly emphasized to participants the very high cost of the fMRI experiment to the relevant participants before the TNT phase. To test this, we performed an independent-sample t-test comparing the total memory checking score in the fMRI sample to that of the behavioral sample. Although the total non-compliance was numerically higher in the behavioral sample, the groups did not differ significantly, *t*(476) = -0.888, *p* > 0.05.

### Most participants expected a final memory test

**Figure 2:**
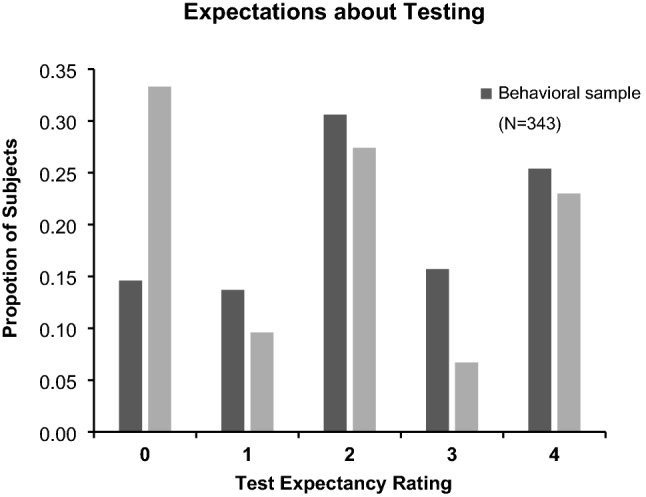
Proportion of participants reporting each level of test expectancy in each of the samples. (0 = not at all; 4 = certain).

We report the test-expectancy data in Fig. 2, with the proportion of participants giving each rating, separately for the fMRI and behavioral samples. The results showed that most participants claimed some suspicion that they would be tested. The mean test expectancy of the overall sample was 2.102 ± 1.426.

Test expectancy was significantly higher in the behavioral sample (2.236 ± 1.357) than it was in the fMRI sample (1.763 ± 1.541), *t*(476) = −  3.300, *p* < 0.001, suggesting that the scanner context may have altered participants’ perceptions of the purpose of the study. Nevertheless, although we sought to characterize the procedure for the TNT experiment as being about attention (rather than memory) before the experiment to all participants, the test expectancy effect still occurred.

### Memory checking is associated with greater test expectancy

**Figure 3:**
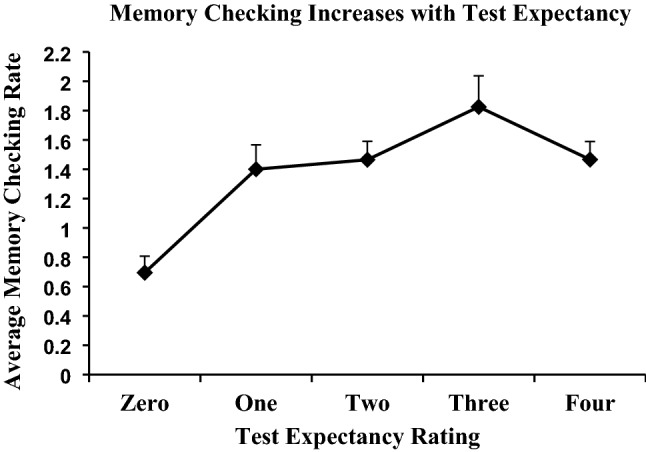
The level of test expectancy observed for each level of memory checking reported in the overall sample. Bars represent + standard errors.

If participants expected a final memory test, they might have been more motivated to check their memory of the learned word pairs during or after No-Think trials. To test this, we computed a Pearson correlation between participants’ test expectancy ratings and their total non-compliance scores (memory checking). Using 1000 bootstrap samples to test for significance, we found a reliable correlation, *r* = 0.185 (95% CI = [0.100, 0.268]), *p* < 0.001. This positive relationship was also significant within the behavioral sample, *r* = 0.196 [0.105, 0.282], *p* < 0.001, but it was only marginally significant in the fMRI sample, *r* = 0.151 [0.002, 0.316], *p* = 0.081. Overall, these results confirmed that memory checking increases when people expect a later test (see Fig. 3).

### Memory checking is associated with reduced suppression-induced forgetting

We tested whether participants’ tendency to check their memories during and after No-Think trials (contrary to the instructions) was associated with the amount of SIF observed on both the SP and IP tests. We also examined which individual behaviors identified in our memory checking questionnaire most strongly moderated SIF. We report the results for both the conditionalized and unconditionalized data below.

### Relationship between memory checking and suppression-induced forgetting

We found that suppression-induced forgetting declined with increasing memory checking. To test this, we first examined the correlation between overall SIF (collapsed over *Test Type*) and memory checking using the unconditionalized data from the full sample, testing for significance based on 1000 bootstrap samples. We observed a significant negative correlation between memory checking and forgetting, *r* = −0.209 (95% CI = [−0.289, −0.135]), *p* < 0.001(see Table 2). This effect was significant individually for the fMRI sample, *r* = -0.236 [−0.377, −0.083], *p* < 0.01, and for the behavioral sample, *r* = -0.199 [−0.285, −0.113], *p* < 0.001. We found a similar overall pattern for the conditionalized data, *r* = −0.162 [−0.249, −0.081], *p* < 0.001. For the fMRI sample, the conditionalized correlation was significant, *r* = −0.177 [−0.335, −0.014], *p* < 0.05, as it was for the behavioral sample, *r* = −0.158 [−0.253, −0.061], *p* < 0.005.

**Table 2:** Correlations between SIF and MCR on the SP and the IP tests.

	n	r	*p*	Bootstrap of 95% CI	
				Lower	Upper
**Experiment 1 (fMRI)**					
IP unconditional	133	−0.203	0.019	−0.350	−0.059
IP conditional	133	−0.171	0.049	−0.317	−0.024
SP unconditional	133	−0.159	0.068	−0.317	0.009
SP conditional	133	−0.090	0.305	−0.252	0.080
**Experiment 2 (behavioral)**					
IP unconditional	343	−0.121	0.025	−0.225	−0.023
IP conditional	343	−0.130	0.016	−0.235	−0.026
SP unconditional	343	−0.199	0.000	−0.293	−0.092
SP conditional	343	−0.163	0.002	−0.255	−0.060
**Full sample**					
IP unconditional	476	−0.146	0.001	−0.225	−0.062
IP conditional	476	−0.142	0.002	−0.227	−0.047
SP unconditional	476	−0.185	0.000	−0.271	−0.100
SP conditional	476	−0.140	0.002	−0.220	−0.057

MCR = Memory Checking Rating; CI = Confidence Interval; bootstrap results are based on 1000 bootstrap samples.

**Figure 4:**
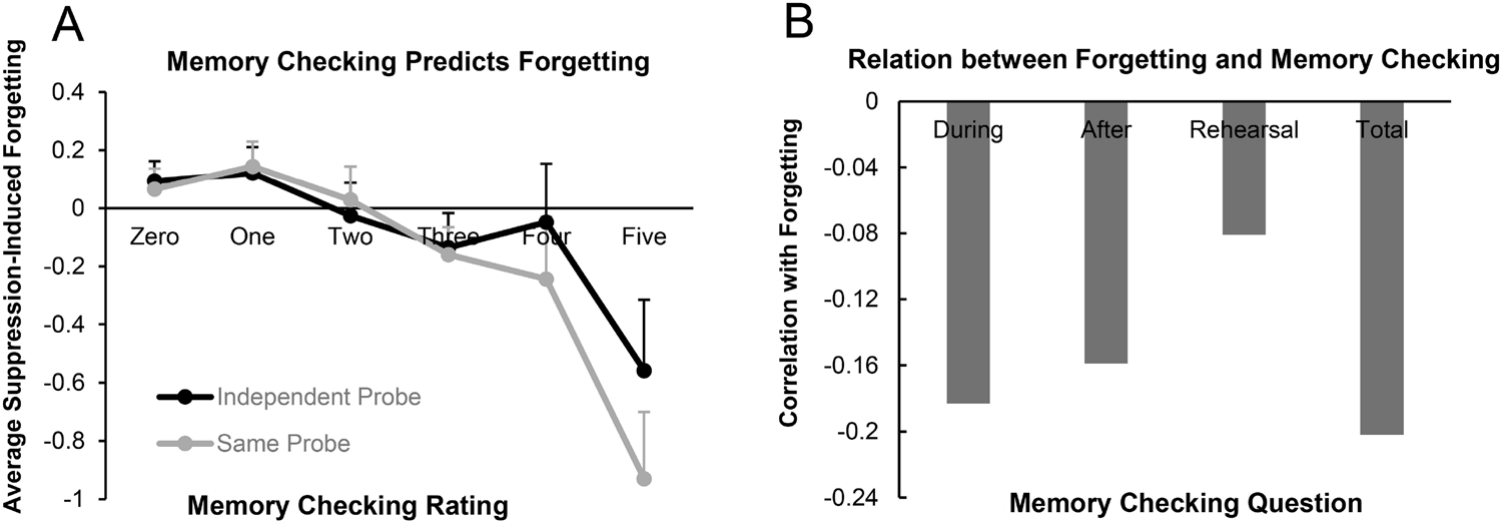
(**A**) The amount of suppression-induced forgetting observed for each level of memory checking reported, separately for the Same-Probe and Independent-Probe tests in the overall sample. Bars represent + standard errors. (**B**) Pearson correlations between SIF and intentional memory checking either during or after No-Think trials, intentional rehearsal of No-Think items, or total non-compliance.

We next separately analyzed the correlation between memory checking and SIF, this time measured separately on the SP test and IP tests (see Fig. 4A). For the SP test, there was a significant negative correlation between SIF and memory checking using the unconditionalized data from the overall sample, *r* = -0.185 [−0.271, −0.098], *p* < 0.001, and also from the behavioral sample alone, *r* = −0.199 [-0.299, −0.098], *p* < 0.001. For the fMRI sample, this correlation was not significant, *r* = −0.159 [−0.322, 0.017], *p* > 0.05. For the conditionalized data, we found a significant negative correlation between SIF and memory checking in the overall sample, *r* = −0.140 [−0.221, −0.058], *p* < 0.005, just as we found in the behavioral sample, *r* = −0.163 [−0.254, −0.062], *p* < 0.005. But, again, this failed to reach significance considering the fMRI sample in isolation, *r* = -0.090 [−0.252, 0.071], *p* > 0.05.

A similar overall pattern of results was observed for the IP test. For the unconditionalized data from the overall sample, SIF was negatively correlated with memory checking, *r* = −0.146 [−0.223, −0.073], *p* < 0.005, a pattern also observed in the separate behavioral, r = −0.121 [−0.215, −0.032], *p* < 0.05, and fMRI samples, *r* = -0.203 [−0.345, −0.051], *p* < 0.05. For the conditionalized data, the negative correlation was similarly significant, though numerically weaker, in the overall sample, *r* = −0.142 [−0.231, −0.063], *p* < 0.005. This negative correlation was also significant in the behavioral sample, *r* = −0.130 [−0.233, −0.026], *p* < 0.05, and in the fMRI sample, *r* = −0.171 [−0.312, −0.013], *p* < 0.05. In sum, the results establish that memory checking is associated with reduced SIF, regardless of test type or data conditionalization.

#### Identifying a memory checking threshold for future studies

Next, we sought to identify a reasonable cutoff score for task compliance to use as an exclusion criterion in future TNT studies. We compared participants’ SIF effect from the overall (combined) sample to zero using a one-sample t-test (separately for the Same Probe and Independent Probe tests) based on participants’ memory checking score (summed across the component items). Significant SIF was observed for the subsamples of participants who had a total memory checking score of either 1, 2, or 3 (lower scores reflect less checking during No-Think trials and, therefore, greater compliance); no SIF was observed when checking scores exceeded 3. This was true of the unconditionalized data from the IP test, as well as for the conditionalized data in the IP test. The same tendency was observed in the unconditionalized data in the SP test, and for the conditionalized data in the SP test. Based on our exploration of the data from our large sample, we would therefore recommend that researchers consider excluding (or considering separately) participants whose total TNT memory checking score is ≥ 4 (see Table 3). We also made Bayesian factor analysis of SIF on the SP and IP tests, according to compliance (see Supplementary Table S1). The results was the same with the one-sample t-test.

**Table 3:** One-Sample t-test of SIF on the SP and IP tests, according to compliance.

	t	df	*p*	Bootstrap^a^ of 95% CI	
				Lower	Upper
**MCR = 0**					
IP unconditional	6.222	172	0.000	0.059	0.110
IP conditional	6.197	172	0.000	0.068	0.126
SP unconditional	6.308	172	0.000	0.071	0.136
SP conditional	6.468	172	0.000	0.075	0.138
**MCR = 1**					
IP unconditional	3.851	121	0.000	0.034	0.098
IP conditional	4.587	121	0.000	0.049	0.124
SP unconditional	6.338	121	0.000	0.084	0.157
SP conditional	7.181	121	0.000	0.103	0.176
**MCR = 2**					
IP unconditional	2.891	84	0.005	0.019	0.103
IP conditional	3.244	84	0.002	0.028	0.122
SP unconditional	3.571	84	0.001	0.038	0.132
SP conditional	4.429	84	0.000	0.065	0.168
**MCR = 3**					
IP unconditional	2.121	56	0.038	0.003	0.103
IP conditional	2.796	56	0.007	0.020	0.125
SP unconditional	2.292	56	0.026	0.007	0.083
SP conditional	3.567	56	0.001	0.031	0.105
**MCR = 4**					
IP unconditional	− 0.023	23	0.982	− 0.067	0.074
IP conditional	0.034	23	0.973	− 0.094	0.106
SP unconditional	0.593	23	0.559	− 0.065	0.130
SP conditional	0.363	23	0.72	− 0.076	0.119
**MCR ≥ 5**					
IP unconditional	0.103	14	0.919	− 0.089	0.093
IP conditional	− 0.189	14	0.853	− 0.128	0.103
SP unconditional	− 3.847	14	0.002	− 0.205	− 0.064
SP conditional	− 2.362	14	0.033	− 0.143	− 0.009

One-sample *t*-tests compared to zero were used to identify when final recall on the various test measures reliably fellow below Baseline (indicating positive suppression-induced forgetting); MCR = Memory Checking Rating (total compliance score across questionnaire items); CI = Confidence Interval; bootstrap results are based on 1000 bootstrap samples.

#### Memory checking during or after a No-Think trial is negatively correlated with suppression-induced forgetting

Although memory checking, overall, was associated with reduced SIF, the preceding analyses left it unclear as to which specific checking behaviors contributed to the observed effect. To examine this, we correlated responses from each item of the memory checking questionnaire with SIF (see Fig. 4B). In the conditionalized recall data, we observed a negative correlation between SIF and both the first item of the questionnaire (“When I saw the red cue word, I quickly checked to see if I remembered the target word.”), *r* = −0.152 [−0.236, −0.072], *p* < 0.005, and with the second question (“After a red cue word went off the screen, I checked to see if I still remembered the target word.”), *r* = −0.129 [−0.223, −0.041], *p* < 0.01. There was not a significant negative correlation between the SIF and the third question (“When I saw a red cue word, I thought about the target word that went with it to purposely improve my memory for that word pair”), *r* = -0.057 [−0.136, 0.027], *p* > 0.05.

A similar pattern was observed for the unconditionalized data (SIF with the first item of the compliance questionnaire, *r* = -0.178 [−0.252, −0.101], *p* < 0.001; SIF with the second item, *r* = −0.173 [−0.260, −0.089], *p* < 0.001; SIF with the third item, *r* = −0.094 [−0.175, −0.009], *p* < 0.05). Thus, these findings show that SIF was lower if people checked their memory for the No-Think item either during or after the trial ended.

#### Test expectancy is not associated with suppression-induced forgetting

Given the correlation between test expectancy and memory checking, one might assume that the test expectancy also predicted the amount of SIF. To our surprise, however, we found a significant correlation between test expectancy and overall SIF in neither the unconditionalized data, *r* = 0.013 [−0.072, 0.100], *p* > 0.05, nor in the conditionalized data, *r* = −0.002 [−0.092, 0.082], *p* > 0.05. This finding held true regardless of whether one examined the relationship between test expectancy and unconditionalized SIF on the SP test, *r* = −0.017 [−0.094, 0.069], *p* > 0.05, or on the IP test, *r* = 0.038 [−0.051, 0.132], *p* > 0.05. Similarly, it did not matter whether it analyzed using the conditionalized data from the SP test, *r* = −0.011 [−0.094, 0.069], *p* > 0.05, or from the IP test, *r* = 0.001[−0.093, 0.095], *p* > 0.05. Clearly, simply expecting a test did not, by itself, reduce SIF. Rather, SIF was moderated only to the extent that such test expectancy led to memory checking behaviors during No-Think trials.

## Discussion

Intuitively, the mnemonic consequences of instructions to suppress retrieval should depend upon whether those instructions actually lead participants to suppress. The number^12^ and quality^37,38^ of the suppression attempts previously have been shown to influence the magnitude of suppression-induced forgetting. However, whereas suppression practice—either in the laboratory or through naturally occurring events^4^—tends to be associated with greater levels of SIF, intentional retrieval practice facilitates recall of the practiced items on standard memory tests. Thus, it makes sense that investigators have reported initial signs that intentionally subverting memory suppression instructions in the TNT paradigm may water down or even reverse SIF^29^. Such observations have encouraged the use of relevant questionnaire-based exclusion criteria in subsequent protocols designed to better focus on the consequences of intentional memory stopping. Here we tested the merits of this concern using two very large samples of healthy young adults participating in the TNT paradigm.

Consistent with previous work^10^, we replicated the canonical below-baseline SIF effect for No-Think items across our two samples using direct suppression instructions. Indeed, SIF arose regardless of whether recall performance was analyzed for all studied items, or only those that were demonstrably learned during the initial study phase. Critically, this evidence for SIF generalized to a test using independent probes. That cue-independent forgetting arose supports the argument that inhibition contributes to SIF^6,10,11,39^.

Relative to SIF, above-baseline facilitation owing to repeatedly practicing the retrieval of Think associates appeared less generalizable to the independent probe variant of the final recall test. No reliable facilitation was detected on the SP test. This result is consistent with previous suggestions that the benefits of rehearsal tend to be most apparent on tests with cues matching those that were originally trained and practiced (i.e., on same probe tests^11,40,41^). Such findings may reflect a feature of the encoding specificity principle: Because the initial encoding process biases the meaning of the items to the original cue, a different final test probe would be expected to reduce recall probability^23,42^. Indeed, the more strongly that a target is associated with its original cue, the more detrimental the effect of shifting cues should be^43^. Our criterion test and measures of Baseline SP recall were consistent with such a strong association having been established through the initial study and test-feedback training. Think items were then subjected to continued practice with the original cue throughout the TNT phase, thereby emphasizing the original bias even further and, presumably, making it especially difficult to retrieve the Think targets given the independent probes.

The primary aim of this study, however, was to determine the extent to which the relative difficulty in recalling associates that participants had been instructed to repeatedly suppress (rather than retrieve) is related to their self-reported level of task compliance and/or test expectancy. Indeed, the data revealed that participants’ self-reported compliance with No-Think task instructions was negatively associated with the magnitude of their SIF effect. Although participants’ memory checking increased when a memory test was expected to occur later in the experiment, test expectancy itself was not directly associated with SIF. The present work is consistent with the concern that task compliance could influence variability in the magnitude of SIF. As such, the present findings indicate that researchers making use of the TNT paradigm should closely monitor participants’ compliance. Moreover, we used a data-driven analysis and found that participants who had a total memory checking score ≥ 4 disproportionately influence the SIF measure, providing a strong rationale for excluding participants meeting this criterion in future studies.

Some might argue that the non-compliance rate in this study is too low for any practical concern, given that we observed reliable SIF in our overall, nevertheless. Indeed, most of our participants reported complying with suppression instructions. However, not all studies making use of the TNT paradigm are able achieve the power afforded by the nearly 500 participants, 48 critical TNT pairs, and 12 repetitions of the critical Think and No-Think cues during the critical phase of the present work. Studies with relatively less power to detect a standard SIF effect would be more sensitive to the distorting effects of non-compliance, even if they undertook all the other measures we employed to ensure understanding of the instructions and shape expectations. Thus, we believe caution is warranted.

Our participants’ overall level of non-compliance was negatively associated with the magnitude of SIF, as were the specific measures of non-compliance (intentionally checking their memory for No-Think associates they had been instructed not to think about) both during and after No-Think trials. Even when test expectancy was at its highest, this negative relationship was still observed. Despite an association between memory checking behaviors and test expectancy, test expectancy itself failed to reliably directly predict the magnitude of SIF on its own, suggesting that task compliance during and around suppression windows is a more powerful determinant of individuals’ memory control scores than are expectations about testing. While expectation of a final test may encourage non-compliant behaviors (e.g., checking one’s memory), many expectant participants apparently were able to resist the urge to do so and go on to demonstrate their control abilities in the form of measurable SIF.

In this, as in other studies of retrieval suppression, several methods were employed to encourage and track participants’ compliance. First, we masked the true focus of the study using a cover story in which participants were told that we were interested in their ability to pay attention and ignore distracting things (e.g., the learned associates whenever cues were presented in red during the main TNT phase). This framing made the avoidance of “distraction” by No-Think items a key goal. To support this framing, we carefully avoided references to “memory” and any hints of a final memory test at all stages of research—from advertisements, to consent forms, to laboratory context (e.g., no memory books on shelves; not memory decor), to instructions or computer displays. By eliminating such references, we avoided encouraging participants to adopt a contraindicated strategy that they might have assumed would improve their retention of No-Think items. Second, we administered a series of diagnostic questionnaires throughout the practice and critical TNT phases to reaffirm the task instructions, correct any apparent misunderstandings, and assess compliance at early stages. These efforts presumably curbed checking behaviors to some extent and contributed to the high level of compliance, overall (i.e., non-compliance would have been even higher, overall, without these procedures in place).

Nevertheless, despite these efforts, the present work documents self-reported evidence that a certain subset of participants still admitted to engaging in some degree of memory-checking behaviors (during and/or after No-Think trials), although deliberate attempts to intentionally rehearse the items were relatively rare. Despite everything, though, we anticipate that some percentage of participants will still fail to comply with the instructions. While no self-report measure is perfect in capturing non-compliance, we believe that our questionnaire, together with an established exclusion threshold (or a covariate entered into the statistical model) could allow researchers to better focus on the aftereffects of actual suppression attempts, if that is their aim. The results of our analyses lead us to conclude that it is critical that compliance be encouraged, measured, and considered during the analysis of suppression-induced forgetting in future studies using the TNT paradigm. Of course, we must acknowledge that there are alternative interpretations of the correlations reported here. It is possible, for example, that participants who were naturally bad at suppressing retrieval sometimes attributed intrusions of the associate as non-compliance (although this contribution was possibly limited by our repeated emphasis that checking needed to be intentional). Alternatively, the correlation between the participants’ compliance and SIF effect might be driven by a third variable that caused both the lack of compliance and poor SIF. Further study, perhaps with manipulations or objective measures to supplement after-the-fact, self-reported compliance scores, the directionality and causality of this observed linkage could be determined.

Researchers intending to examine the aftereffects of retrieval suppression using the TNT paradigm may gain substantial added traction and clarity by focusing on participants who are naturally motivated to comply with the instructions; over and above that, though, efforts to foster a higher level of intrinsic motivation in participants to suppress the targets stand to increase the efficiency of this research and potentially translate to real-world applications. Variants of the paradigm incorporating negatively valanced or personally relevant No-Think items may represent one means to this end. Other approaches may involve increasing the stakes of a distraction popping to mind, perhaps by tying online success measures to reward (or failures to low-grade but salient forms of punishment).

## Conclusion

In conclusion, we reported the results from two large, independent samples of healthy participants” demonstrating that access to encoded memories was reliably impaired through retrieval suppression prompted by the TNT paradigm. Importantly, the results further revealed that participants’ self-reported compliance with the suppression instructions—but not test expectancy—predicted SIF. As such, the current study provides clear evidence consistent with task compliance being a likely source of variability in the SIF, as measured via the TNT paradigm. To better isolate the forgetting effect of interest, future TNT studies should ensure participants’ compliance with the suppression instructions through the careful administration of task instructions and regular use of diagnostic questionnaires. Moreover, strategies designed to improve participants’ intrinsic motivation to suppress the memory could be considered. Directions to suppress, on their own, may not always be sufficient to induce forgetting, especially when participants may be motivated to retain the suppressed content (as when they expect a later test). Given the proper commitment to push unwanted memories out of mind, suppression reliably yields suppression-induced forgetting.

## Supplementary Information

Supplementary Information.
