# Supplementary material for: Task compliance predicts suppression-induced forgetting in a large sample
Source: Sci Rep. 2021 Oct 11;11:20166. doi: 10.1038/s41598-021-99806-8 (PMC8505621; doi:10.1038/s41598-021-99806-8)
Supplement: Supplementary file 1 — Supplementary Information. [file 41598_2021_99806_MOESM1_ESM.docx]

| **MCR=0** | N | Mean | SD | BF₁₀ |
| --- | --- | --- | --- | --- |
| IP unconditional | 173 | 0.083 | 0.176 | 5.025e +6 |
| IP conditional | 173 | 0.096 | 0.205 | 4.421e +6 |
| SP unconditional | 173 | 0.102 | 0.212 | 7.716e +6 |
| SP conditional | 173 | 0.104 | 0.212 | 1.746e +7 |
| **MCR=1** |  | |  |  |
| IP unconditional | 122 | 0.066 | 0.189 | 188.871 |
| IP conditional | 122 | 0.088 | 0.213 | 2684.567 |
| SP unconditional | 122 | 0.12 | 0.209 | 4.865e +6 |
| SP conditional | 122 | 0.139 | 0.214 | 2.827e +8 |
| **MCR=2** |  |  |  |  |
| IP unconditional | 85 | 0.064 | 0.204 | 11.403 |
| IP conditional | 85 | 0.078 | 0.222 | 29.376 |
| SP unconditional | 85 | 0.087 | 0.226 | 76.219 |
| SP conditional | 85 | 0.116 | 0.242 | 1253.037 |
| **MCR=3** |  |  |  |  |
| IP unconditional | 57 | 0.054 | 0.192 | 2.249 |
| IP conditional | 57 | 0.074 | 0.2 | 9.597 |
| SP unconditional | 57 | 0.043 | 0.142 | 3.158 |
| SP conditional | 57 | 0.068 | 0.143 | 70.85 |
| **MCR=4** |  |  |  |  |
| IP unconditional | 24 | -8.333e-4 | 0.175 | 0.211 |
| IP conditional | 24 | 0.002 | 0.241 | 0.22 |
| SP unconditional | 24 | 0.029 | 0.241 | 0.358 |
| SP conditional | 24 | 0.018 | 0.248 | 0.289 |
| **MCR≥5** |  | | |  |
| IP unconditional | 15 | 0.005 | 0.175 | 0.284 |
| IP conditional | 15 | -0.011 | 0.219 | 0.23 |
| SP unconditional | 15 | -0.137 | 0.138 | 0.077 |
| SP conditional | 15 | -0.081 | 0.132 | 0.095 |
| Note: MCR= Memory Checking Rating; For all tests, s specifies that the population mean is greater than 0. | | | | |

Table S1. Bayesian factor analysis of SIF on the SP and IP tests, according to compliance.
